# Amebic Liver Abscess: An Unusual Cause of Budd–Chiari Syndrome

**DOI:** 10.1155/crdi/1133906

**Published:** 2025-12-15

**Authors:** Saran Lal Ajai Mokan Dasan, Ramesh R.

**Affiliations:** ^1^ Department of Internal Medicine, Velammal Medical College Hospital and Research Institute, Madurai, 625009, India, velammalmedicalcollege.edu.in

**Keywords:** amoebic liver abscess, ascites, Budd–Chiari syndrome, diabetes mellitus, pleural effusion, vascular thrombosis

## Abstract

Amebiasis is a significant public health issue in tropical regions, with liver abscess as its most common extraintestinal complication. Budd–Chiari syndrome secondary to amoebic liver abscess is rare and seldom reported. We present a 37‐year‐old man with poorly controlled Type 1 diabetes who developed fever, abdominal pain and distension. Imaging identified a left lobe liver abscess with hepatic vein and inferior vena cava thrombosis, consistent with Budd–Chiari syndrome. The patient received image‐guided drainage, broad‐spectrum antimicrobials, and anticoagulation, leading to clinical improvement and vascular recanalization. We intend to bring to notice, the need to consider hepatic venous outflow obstruction in patients with amoebic liver abscess and ascites or lower extremity edema. Early diagnosis and combined medical and interventional management can help prevent irreversible liver damage.

## 1. Introduction

Amebiasis, caused by *Entamoeba histolytica*, continues to be a significant parasitic infection in developing regions, resulting in approximately 40 million infections and nearly 100,000 deaths each year worldwide [1]. The liver is the most frequent extraintestinal site of involvement, resulting in amoebic liver abscess (ALA), a condition characterized by necrotic hepatocellular destruction secondary to trophozoite invasion. While ALA classically presents with fever, right‐upper‐quadrant pain, and hepatomegaly, its vascular complications remain under‐recognized. Recent radiological series have shown that hepatic or portal venous thrombosis may accompany up to 5.6% cases, yet clinically significant hepatic venous outflow obstruction—manifesting as Budd–Chiari syndrome (BCS)—is exceedingly rare [2]. We report a rare case of BCS secondary to a left‐lobe ALA in a patient with poorly controlled Type 1 diabetes mellitus.

## 2. Case Details

**Figure 1:**
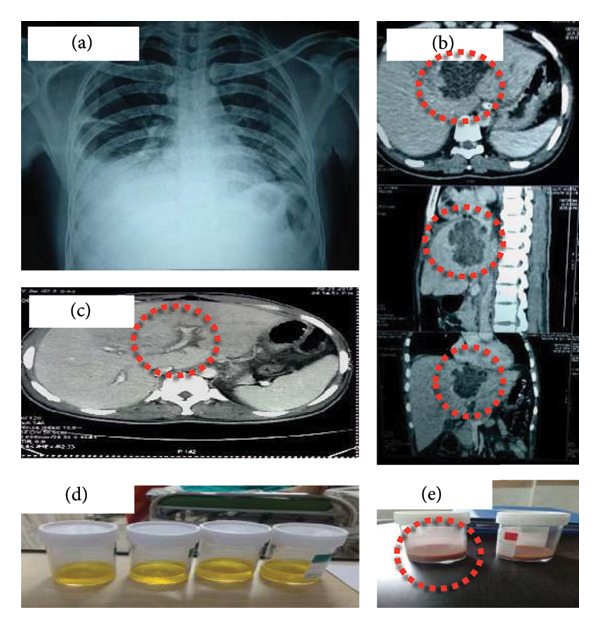
(a) Chest radiograph (posteroanterior view) showing a moderate right‐sided pleural effusion, likely reactive or due to *trans*‐diaphragmatic extension from the adjacent hepatic abscess. (b) Contrast‐enhanced CT of the abdomen demonstrating a peripherally enhancing, centrally hypodense lesion measuring 7.2 × 5.3 × 7.5 cm in the left hepatic lobe, consistent with an amoebic liver abscess. (c) Contrast‐enhanced CT (axial view) showing hypodense filling defects in the middle and left hepatic veins and the suprahepatic segment of the inferior vena cava, indicating hepatic venous thrombosis compatible with Budd–Chiari syndrome. (d) Diagnostic paracentesis revealing straw‐colored ascitic fluid, later confirmed to be exudative on biochemical analysis (elevated protein and LDH). (e) Ultrasound‐guided percutaneous drainage of the liver abscess demonstrating evacuation of thick reddish‐brown pus with the characteristic “anchovy‐sauce” appearance typical of amoebic infection.

A 37‐year‐old male with medical history of insulin‐dependent diabetes mellitus presented to emergency with fever, abdominal pain, and distension for 5 days. He also reported a few episodes of diarrhea 2 weeks ago which has since resolved but continues to experience high colored urine, lower extremity swelling, and SOB. Since then, the patient also had poorly controlled diabetes mellitus due to nonadherence to insulin therapy. On admission, he had tachypnea, tachycardia, and hypotension. On examination, he was pale, icteric with bilateral pitting pedal edema. Auscultation revealed decreased air entry on right lower lung fields with bilateral basal crepitations. On abdominal examination, there was right hypochondrial and epigastric tenderness extending to the left hypochondrium. The liver was palpable 3 cm below the right costal margin with tenderness more pronounced over the left side corresponding to the abscess site. He was initially treated with IV fluids, Nasal O_2_, analgesics, and empiric antibiotics. X‐ray revealed a right sided pleural effusion (Figure 1(a)), and CT abdomen revealed left liver abscess with filling defect in hepatic veins and IVC with moderate ascites (Figures 1(b) and 1(c)). Diagnostic peritoneal taping was done which showed straw colored ascitic fluid (Figure 1(d); Table 1).

**Table 1:** Baseline hematologic, biochemical, and ascitic fluid parameters at presentation, demonstrating leukocytosis, elevated hepatic enzymes, hyperbilirubinemia, and hypoalbuminemia, consistent with acute inflammatory hepatic insult.

Lab parameter	Patient value	Reference range
Hemoglobin	9.2 g/dL	13–17 g/dL
Total count	41,000 cells/mm^3^	4000–11000 cells/mm^3^
Platelet count	2.2 × 10^5^ cells/mm^3^	1.5–4.5 × 10^5^ cells/mm^3^
ESR (1 h)	89 mm	< 20 mm
Blood urea	47 mg/dL	10–50 mg/dL
Creatinine	1.3 mg/dL	0.6–1.2 mg/dL
Random plasma glucose	68 mg/dL	70–140 mg/dL
HbA1c	12%	< 5.7%
Total bilirubin	2.68 mg/dL	0.3–1.2 mg/dL
Direct bilirubin	2.03 mg/dL	< 0.3 mg/dL
Total protein	5.1 g/dL	6–8 g/dL
Albumin	2.7 g/dL	3.5–5 g/dL
AST	11 IU/L	10–40 IU/L
ALT	75 IU/L	7–56 IU/L
ALP	438 IU/L	44–147 IU/L
PT/INR	24 s	11–15 s
PTT	35 s	25–35 s
Stool examination	No ova/cyst/tropozoite seen	No ova/cyst/tropozoite seen
Ascitic fluid analysis		
LDH	308 IU/L	
Protein	2.9 g/dL	
Glucose	131 mg/dL	
*Entamoeba histolytica* antibodies (EIA)	7.25	< 0.9

Abscess drainage was planned under USG guidance, but the procedure was aborted due to excess bleeding, and the patient was transfused with 4 units of FFP and Vitamin K. Abscess drainage done successfully the next day with reddish‐brown colored (Figure 1(e)) purulent collection sent for culture/sensitivity, Gram staining, and wet mount. A pig‐tail catheter was inserted into the abscess cavity to prevent recollection. No trophozoites/bacterial growth could be demonstrated from the pus. Serological testing was positive for Entamoeba histolytica antibodies. JAK‐2 mutation done as part of thrombotic workup turned out negative.

Differentiating amoebic from pyogenic liver abscess is essential as management differs significantly. In this patient, culture of aspirated pus showed no bacterial growth, and Gram staining was negative. Serology for *Entamoeba histolytica* antibody was positive by ELISA, which, in conjunction with the characteristic “anchovy‐sauce” aspirate, strongly supports amoebic etiology. Although PCR‐based or antigen detection assays provide higher specificity, these modalities were unavailable in our setting. The absence of polymicrobial culture, lack of biliary pathology, and favorable response to metronidazole further excluded a pyogenic source or mixed infection.

**Figure 2:**
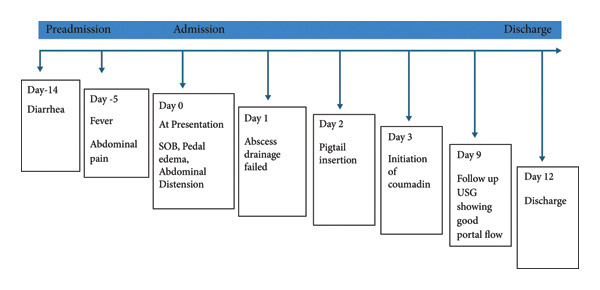
Clinical timeline summarizing the sequence of presentation, intervention, and recovery.

We treated him with intravenous fluids, metronidazole 750 mg TID for 10 days, followed by diloxanide furoate 500 mg for 10 days, subcutaneous insulin, heparin bridged to warfarin with PT/INR monitoring. Anticoagulation was initiated on Day 3 after stabilization from the drainage‐related bleeding episode. The patient responded to the treatment. He developed no further complications during hospital stay. Follow‐up ultrasound, done at the 9th day, showed recanalized flow in middle and left hepatic vein and partial flow in suprahepatic portion of IVC. The patient was discharged on the 12th day of admission. We started our patient on anticoagulants only on the 4th day of diagnosis owing to the bleeding complication developed during the time of abscess drainage. He was continued on oral anticoagulants for 6 months following which USG Doppler showed complete recanalization (Figure 2).

## 3. Discussion

Amebiasis, caused by *E. histolytica*, is a protozoal infection primarily affecting the gastrointestinal tract. Although many infected individuals remain asymptomatic, a small proportion may develop invasive disease, including amoebic colitis and extraintestinal manifestations such as liver abscesses. The progression from intestinal colonization to liver involvement is believed to occur via hematogenous spread through the portal vein. However, active gastrointestinal symptoms are often absent at the time of liver abscess diagnosis.

The most common extraintestinal manifestations of amebiasis are ALA, which usually involves the right lobe of the liver due to differential portal blood supply [1, 3]. There is a male preponderance with alcohol use being another strong risk factor [1]. Liver abscess is a common complication affecting 9% of cases with 18% mortality if the abscess ruptures [4, 5]. The overall inpatient mortality rate of patients with ALA was at 1.1% [2]. CT is the recommended modality for diagnosing liver abscess. Holversan et al. described the classical appearance of various types of liver abscess with amoebic abscesses most often presenting as solitary, low‐density areas in the right lobe of the liver. However, in individual cases, they are indistinguishable from pyogenic abscesses. Microbiologic or immunologic confirmation is necessary for the diagnosis [6].

### 3.1. Diagnostic and Therapeutic Considerations

The definitive diagnosis of amoebic colitis is made by the demonstration of hematophagous trophozoites in the stool. Diagnosis of invasive amebiasis can be difficult, as trophozoites are rarely present in the aspirated abscess material due to their localization at the abscess margins rather than the necrotic center. As a result, serological testing plays a critical role in confirming the diagnosis. Antibody detection by ELISA detects antibody specific for *E. histolytica* in approximately 95% of patients with 100% sensitivity and 99.1% specificity in patients with ALA [1]. If antibodies are not detectable in patients with an acute presentation of suspected ALA, a second specimen can be drawn 5–7 days later. Serological testing can remain elevated for months, and as such in patients from endemic areas, PCR can detect circulating DNA of ALA patients with 89.5% sensitivity and 100% specificity [1].

Treatment of ALA involves using both tissue amebicides and extraluminal amebicides. Metronidazole 500–750 mg PO TID for 7–10 days or tinidazole 2g PO once or ornidazole 2g PO once can be given as tissue amebicide. Diloxanide furoate 500 mg PO TID for 20 days or paromomycin 500 mg PO TID for 7 days can be given as luminal amebicide [1, 7]. Failure to eradicate Entamoeba with luminal amebicide can result in relapse.

Not all cases of ALA require abscess drainage. Gibney et al. in 1990 described the indication of abscess drainage which has since been revised [8]. The current indications for abscess drainage are to rule out pyogenic abscess (particularly in multiple abscess), lack of clinical response with 3–5 days of medical management, threat of imminent rupture, pregnant patients, and in left lobe abscess to prevent rupture into pericardium [9]. Though some have achieved remission with just medical therapy [7], drainage might provide confirmation and symptom relief. As more cases of hepatic thrombosis emerge, indications for abscess drainage might change, though anticoagulant is the main stay of treatment for hepatic thrombosis, drainage would remove the nidus for subsequent thrombus formation, and as such vascular thrombus is recommended as an indication for percutaneous drainage by some researchers [10].

### 3.2. Vascular Complications and Literature Review

The literature on association of ALA with vascular complications is limited. BCS is a hepatic outflow obstruction which can involve one or more hepatic vein, rarely involving IVC and right atrium with symptoms of hepatic and downstream vascular congestion. Autopsy done on fatal cases of amoebic abscess were the first to report venous thrombosis in ALA [10]. Aikat et al. in a study of 79 cases of fatal hepatic amebiasis, demonstrated PVT and hepatic vein thrombosis (HVT) in 29% and 27%, respectively [11]. The study by Priyadarshi et al. found 43 (69%) patients of liver abscess with venous thrombosis as determined by multiphase CT imaging, though not all these patient had manifestations of Budd–Chiari [10]. Sodhi et al. have reported a similar case of ALA with vascular thrombosis as ours [12]. Arora et al. reported a case of ALA with thrombus extending to IVC who had an uneventful recovery with abscess drainage, antimicrobials, and anticoagulation [13]. Though vascular thrombosis appears to be much prevalent, the reported incidence of symptomatic occlusion in the form of Budd–Chiari is seldom reported. The literature review only revealed very few such cases [14–16]. Makthula et al. reported a case of IVC and HVT in a patient with ALA and with clot extension to right atrium and pulmonary vasculature owing to abscess rupture [17].

The pathogenesis of thrombosis in ALA is multifactorial. Traditionally, amoebic emboli from the colon are thought to occlude small hepatic venules, initiating clot formation [10]. Other proposed theory is endothelial hyperactivation from prolonged exposure to amoebic toxins that triggers thrombosis. In addition, mechanical compression of the inferior vena cava or hepatic veins by the abscess disrupts laminar flow (Virchow’s triad) and induces stasis [17]. The ensuing local inflammation, coagulopathy, and contiguous spread of infection to the vessel wall promote endotheliitis and thrombus propagation into the hepatic veins and IVC.

Owing to the rarity, medical management of it is not well documented, and the duration of anticoagulants is determined empirically due to the lack of strong evidence. Our case underscores the importance of maintaining a high index of suspicion for vascular complications in patients with ALA and supports early drainage in appropriate clinical settings to mitigate thrombotic risk.

## 4. Conclusion

Although BCS is a rare complication of ALA, it should be considered in patients presenting with hepatomegaly, ascites, pedal edema, or signs of hepatic venous congestion. Early diagnosis through appropriate imaging and laboratory workup is essential. Image‐guided abscess drainage, when feasible, can not only aid in source control but also may also reduce thrombotic risk by relieving local vascular compression. In selected cases, anticoagulation may support vascular recanalization. Clinicians should maintain a high degree of vigilance for vascular involvement in cases of ALA, particularly those with atypical presentations or severe systemic features.

## Ethics Statement

This case report was conducted in accordance with the ethical standards of the institutional and national research committees and with the 1964 Helsinki Declaration and its later amendments.

## Consent

Informed consent was obtained from the patient for publication in scientific medical journals.

## Conflicts of Interest

The authors declare no conflicts of interest.

## Funding

No external funding was reported by any of the authors.

## Data Availability

All relevant clinical, imaging, and histopathological data supporting the findings of this case report are included within the article. No additional datasets were generated or analyzed. Deidentified supporting material (including radiological and histopathological images) may be made available from the corresponding author upon reasonable request, in accordance with patient‐privacy policies.
